# Mesenteric Inflammatory Venoocclusive Disease in a Patient with Sjögren's Syndrome

**DOI:** 10.1155/2014/423420

**Published:** 2014-11-16

**Authors:** Raquel Rios-Fernández, José-Luis Callejas-Rubio, Mercedes Caba-Molina, Rosa Ríos-Peregrina, Norberto Ortego-Centeno

**Affiliations:** ^1^Autoimmune Systemic Diseases Unit, Hospital Clínico San Cecilio, C/Dr. Olóriz s/n, 18012 Granada, Spain; ^2^Department of Pathology, Hospital Clínico San Cecilio, C/Dr. Olóriz s/n, 18012 Granada, Spain

## Abstract

Mesenteric inflammatory venoocclusive disease is an uncommon cause of intestinal ischemia. Certain diseases, such as hypercoagulation disorders, autoimmune diseases, or drugs have been associated with the pathogenesis of mesenteric inflammatory venoocclusive disease. Here, we report a patient with Sjögren's syndrome who underwent surgery for suspected acute appendicitis with a subsequent pathological diagnosis of mesenteric inflammatory venoocclusive disease.

## 1. Introduction

Mesenteric inflammatory venoocclusive disease (MIVOD) is an uncommon cause of intestinal ischemia affecting the veins of the bowel and mesentery, which causes thrombotic occlusion of these veins. The etiology of MIVOD remains unclear because it is rarely suspected and often underreported.

The occlusion of mesenteric veins and their tributaries can be associated with various conditions, for example, hypercoagulation disorders, Behçet's disease, systemic lupus erythematosus, and eosinophilic granulomatosis with polyangiitis (Churg-Strauss syndrome) [1–5]. Certain drugs have also been implicated as etiological agents, for example, rutoside, reserpine, methyldopa, and amiloride [6]. In a series of cases with mesenteric venous thrombosis, no predisposing cause was determined [7].

Here we report a patient with Sjögren's syndrome (SS) who underwent surgery for suspected acute appendicitis with a subsequent pathological diagnosis of MIVOD. To our knowledge, this is the first case of MIVOD in a patient with SS, illustrating another possible gastrointestinal manifestation of this syndrome.

## 2. Case Report

A 60-year-old woman, smoker (20 cigarettes/day), with a history of SS, biliary cirrhosis, hypertension, hypercholesterolemia, and osteoporosis presented to the emergency department experiencing initial epigastric pain accompanied by progressive pain in the right lower quadrant, nausea, and fever over the last 12 days. At the time the patient was taking budesonide, ursodeoxycholic acid, enalapril, alendronate, and atorvastatin. She had undergone surgery for carpal tunnel syndrome and since then was taking NSAIDs.

On physical examination, she was normotensive and body temperature was 38°C. She was found to have lower right quadrant tenderness with peritoneal signs. Bowel sounds were normal.

Laboratory tests showed high C-reactive protein levels (1.80 mg/dL; n.v. < 0.05 mg/dL). White blood cell count and liver and pancreatic enzyme values were within normal ranges. The following results were obtained with autoantibody testing: positive anti-nuclear antibody was identified at a titer of 1 : 320 with a speckled pattern and the anti-Ro/SS-A Ab value was 717 U/mL (n.v. < 10 U/mL). Other autoantibodies, including anti-La/SS-B Ab, IgM rheumatoid factor, anti-double-stranded DNA, anti-RNP, anti-Scl-70, anti-Jo-1, anti-centromere, IgM aCL, IgG aCL, and lupus anticoagulant, were negative. Coagulation tests were normal.

The abdominal ultrasound showed an ascendant intestinal loop with 14 mm abnormal wall thickening surrounded by fluid and hepatic steatosis.

The initial clinical impression was that of acute appendicitis and a decision to operate was made. The laparotomy revealed mesenteric congestion of the right colon with widening of the terminal ileum. A normal appendix was removed and a mesocolon biopsy was undertaken.

**Figure 1:**
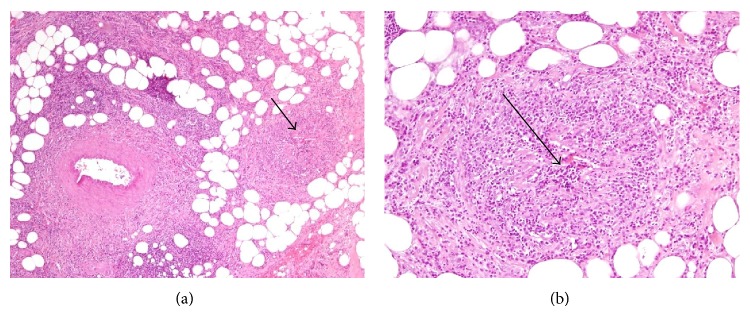
(a) Adipose tissue with heterogeneous intense inflammatory infiltrate consisting of lymphocytes and plasma cells polytypic. The inflammatory infiltrate around the artery but infiltrates vein walls (arrow). H-E original magnification ×40. (b) Higher magnification, a vein totally occluded (arrow). H-E original magnification ×100.

Microscopic examination revealed marked edema and a perivenular lymphocytic infiltrate with some veins totally occluded. The arteries and arterioles were normal. These histopathological findings are consistent with the diagnosis of MIVOD (Figure 1).

The patient had no postoperative complications and recovered completely; no recurrence was observed during the followup period. Blood tests for hypercoagulability and systemic vasculitis were negative.

## 3. Discussion

Sjögren's syndrome (SS) is an autoimmune exocrinopathy involving mainly the parotid and lacrimal glands, although it can involve almost any other part of the gut. The degree to which SS affects the small and large bowel is unclear. Other symptoms include abdominal discomfort in 0%–37% of the cases, nausea in 0%–5%, constipation in 0%–23%, diarrhea in 0%–9%, and malabsorption in 0%–5%. However, documented intestinal involvement is rare to absent in large series [8].

There are few reported cases of SS associated with inflammatory bowel disease, Crohn's disease, or ulcerative colitis [9, 10]. SS has also been linked with pneumatosis cystoides intestinalis [11], colon cancer [12, 13], protein-losing gastroenteropathy [14], and intestinal pseudoobstruction [15]. Involvement of internal organs is more likely if the vasculitis is associated with cryoglobulins [16].

A distinctive feature of MIVOD is the vasculitis of the mesenteric veins and their intramural tributaries. MIVOD seems to affect the colon in more than 50% of the patients, although it has also been reported to affect the small bowel, omentum, and gallbladder [17]. It is characterized by mesenteric venous inflammation and thrombosis, which results in chronic ischemia. No involvement of arterial inflammation or occlusion has been described.

Due to discrepancies in histological and imaging findings, these cases constitute a diagnostic challenge.

Inflammatory bowel disease, intestinal ischemia, and acute appendicitis are the major differential diagnosis. It is important to distinguish MIVOD from mesenteric venous thrombosis, as these patients may be put on anticoagulant therapy. Lymphocytic, necrotizing, and granulomatous patterns of inflammation in the mesenteric veins are characteristic features of MIVOD, not found in mesenteric venous thrombosis.

MIVOD is a pathological diagnosis; surgery is required for a definitive diagnosis and decide on the therapeutic procedure. Maintenance therapy is not necessary since the prognosis of MIVOD is excellent. Only one case of recurrent MIVOD has been reported [18]. Our patient recovered completely, and there has been no recurrence in 2 years of followup.
